# Errata

**DOI:** 10.1200/JCO.2016.71.6407

**Published:** 2017-01-10

The October 10, 2016, article by Ganzel, et al, entitled “Extramedullary Disease in Adult Acute Myeloid Leukemia Is Common but Lacks Independent Significance: Analysis of Patients in ECOG-ACRIN Cancer Research Group Trials, 1980-2008” (J Clin Oncol 34: 3544-3553, 2016), contained an error.

Peter H. Wiernik was omitted as an author on this manuscript. He should have been listed as the second to last author in the byline, between Dr Cripe and Dr Tallman. The byline should have read as: Chezi Ganzel, Judith Manola, Dan Douer, Jacob M. Rowe, Hugo F. Fernandez, Elisabeth M. Paietta, Mark R. Litzow, Ju-Whei Lee, Selina M. Luger, Hillard M. Lazarus, Larry D. Cripe**, Peter H. Wiernik** and Martin S. Tallman.

His affiliation is at the **St. Luke's-Roosevelt Medical Center**.

His contributions are **Collection and assembly of data, manuscript writing and final approval of manuscript.**

His conflict of interest information is provided below.

**Honoraria**

**TRACON Pharma, Novartis**

**Speakers' Bureau**

**Novartis**

**Travel, Accommodations, Expenses**

**Novartis**

The online version has been corrected in departure from the print. The authors apologize for the error.

